# Total Hip Arthroplasty in a Patient with Oto-Spondylo-Megaepiphyseal Dysplasia Planned by Three-Dimensional Motion Analyses and Full-Scale Three-Dimensional Plaster Model of Bones

**DOI:** 10.1155/2018/8384079

**Published:** 2018-01-23

**Authors:** Takeyuki Tanaka, Hideya Ito, Hirofumi Oshima, Nobuhiko Haga, Sakae Tanaka

**Affiliations:** ^1^Department of Orthopaedic Surgery, Faculty of Medicine, The University of Tokyo, 7-3-1 Hongo, Bunkyo-ku, Tokyo 113-8655, Japan; ^2^Bone and Joint Orthopaedic Surgery, Japanese Red Cross Medical Center, 4-1-22 Hiroo, Shibuya-ku, Tokyo 150-8935, Japan; ^3^Department of Rehabilitation Medicine, Graduate School of Medicine, The University of Tokyo, 7-3-1 Hongo, Bunkyo-ku, Tokyo 113-8655, Japan

## Abstract

We present the case of a 28-year-old woman with oto-spondylo-megaepiphyseal dysplasia, which is a rare skeletal disorder, who underwent bilateral total hip arthroplasty. Full-scale three-dimensional plaster model of the acetabulum and the femur provided us with a feasible preoperative plan. Pre- and postoperative three-dimensional motion analyses proved a significant improvement in her ambulation and confirmed the efficacy of total hip arthroplasty. In conclusion, full-scale three-dimensional plaster models of the bone and three-dimensional motion analyses were useful for total hip arthroplasty in patients with skeletal dysplasia.

## 1. Introduction

Oto-spondylo-megaepiphyseal dysplasia (OSMED) is a rare skeletal disorder characterized by a flat midface, severe sensorineural hearing loss, and early-onset osteoarthritis. Cleft palate and micrognathia are also common findings [1]. Researchers have identified gene abnormalities related to type II and type XI collagen [2, 3]. Although about 30 patients have been reported in the literature [2, 4–6], there is a paucity of published data regarding the treatment of locomotive dysfunctions.

Total hip arthroplasty (THA) is an established modality of treatment for patients with osteoarthritis of the hip joint, and preoperative planning is mandatory for preparing optimal implants [7]. Although the radiographic templating techniques achieve satisfactory results in most cases [8, 9], they are not always applicable to patients with unusual skeletal deformities. Moreover, multiple involvements of the joints make it difficult to assess the contribution of the hip joints to gait disturbance. However, recent advancements in computer technology could help orthopedic surgeons not only to plan but also to evaluate the efficacy of hip surgery in these patients [10, 11]. The case of a 28-year-old woman with OSMED who suffered from multiple joint deformities, including bilateral hip joints, is presented.

## 2. Case Presentation

A 28-year-old woman was referred for a gait disturbance with reduced range of motion (ROM) of bilateral hip joints and bilateral hip pain. At the age of 2 years, she had been diagnosed as having bilateral hearing loss and had undergone surgery to correct a cleft palate. From the age of 20 years, she had noticed stiffness of the hip joints and difficulty in walking. At the age of 23 years, clinical geneticists had diagnosed her as having OSMED and identified a COL2A1 mutation relating to type II collagen.

**Figure 1:**
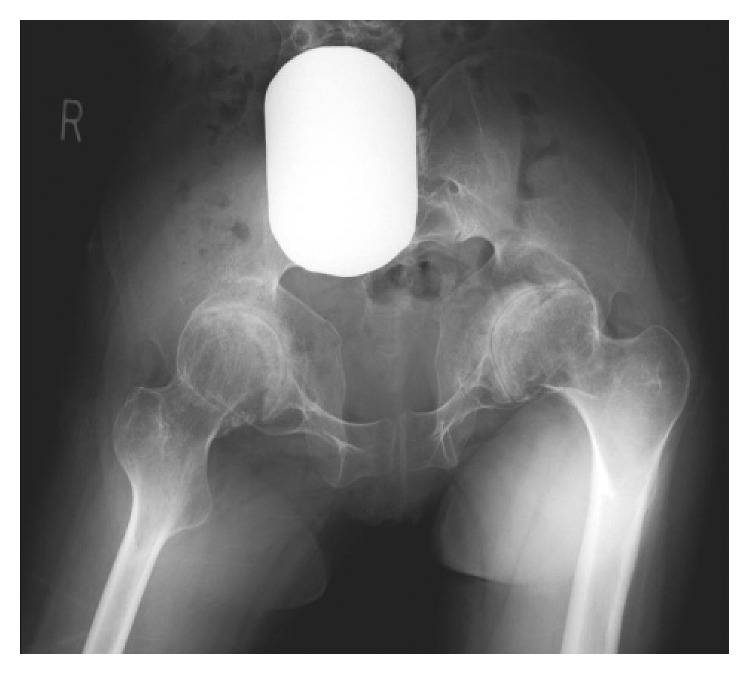
Preoperative radiograph showing end-stage osteoarthritis of the hip joints associated with large femoral heads.

**Figure 2:**
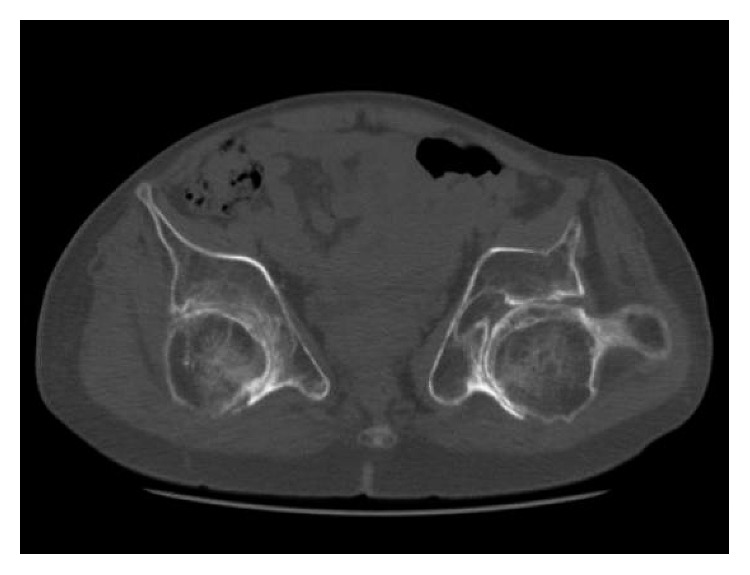
Axial CT image of the hip joints, demonstrating significant retroversion of the acetabulum.

**Figure 3:**
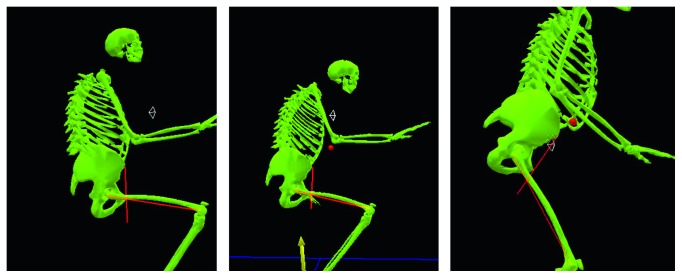
Preoperative three-dimensional motion analysis demonstrates fixed anterior tilting of the pelvis, limited mobility of the lumbar spine, and anterior deviation of the center of gravity.

At the time of the referral, although she could walk for a few minutes using 2 Lofstrand crutches, she used a wheelchair in her daily life. Her height was 155 cm (−0.1 SD), and her weight was 47 kg (−0.8 SD). Physical examination revealed significantly reduced ROM in the limb joints and spine; ROM of both hip joints showed 60° of flexion contracture, 80° of flexion, 0° of abduction, and 10° of adduction. The Harris Hip Score (HHS) [12] was 19 on the right and 20 on the left. Radiographs showed multiple changes of the skeleton, including end-stage osteoarthritis of the hip joints (Figure 1) and knee joints and hyperlordosis of the lumbar spine. The computed tomography (CT) images of the hip joints revealed significant retroversion of the acetabulum (Figure 2). A three-dimensional motion analysis system (VICON MX, SGI, Tokyo, Japan) was used to analyze her standing up and her gait; fixed anterior tilting of the pelvis and limited mobility of the lumbar spine were demonstrated. Furthermore, it also revealed that her center of gravity deviated anteriorly because she could not compensate for severe flexion contractures of bilateral hip joints due to limited mobility of the lumbar spine. Therefore, it was possible to identify that hip dysfunction had a major impact on gait disturbance (Figure 3).

**Figure 4:**
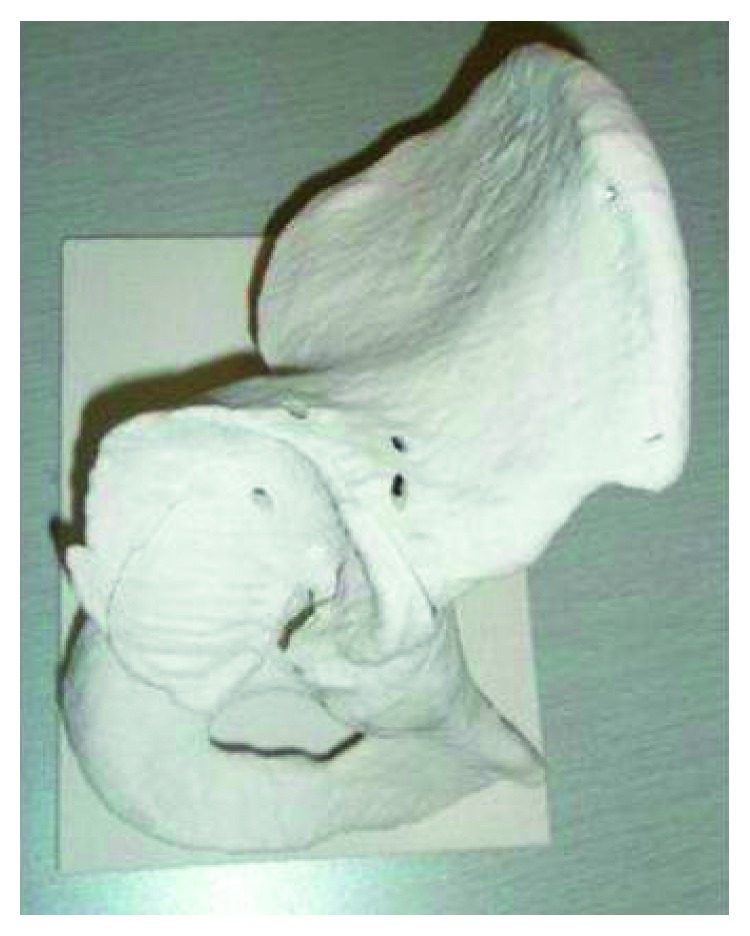
Full-scale three-dimensional plaster models of the pelvis, showing the aspherical acetabular cavity and the thin acetabular wall.

**Figure 5:**
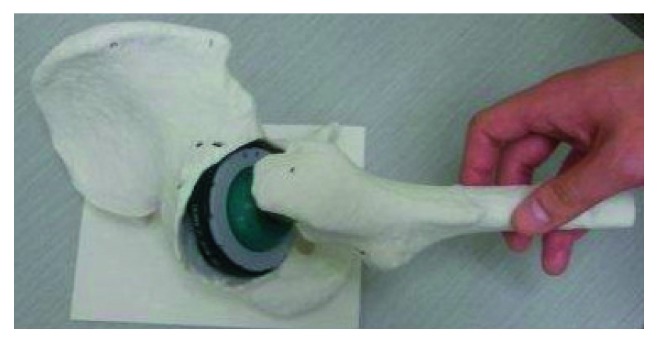
Setting a 68-millimeter diameter hemisphere at 0° of anteversion on the plaster model. This demonstrates that its posterior part projects backward from the acetabulum and its anterior part is deep in the bony wall. To avoid bony impingement, a significant amount of the bone needs to be removed.

**Figure 6:**
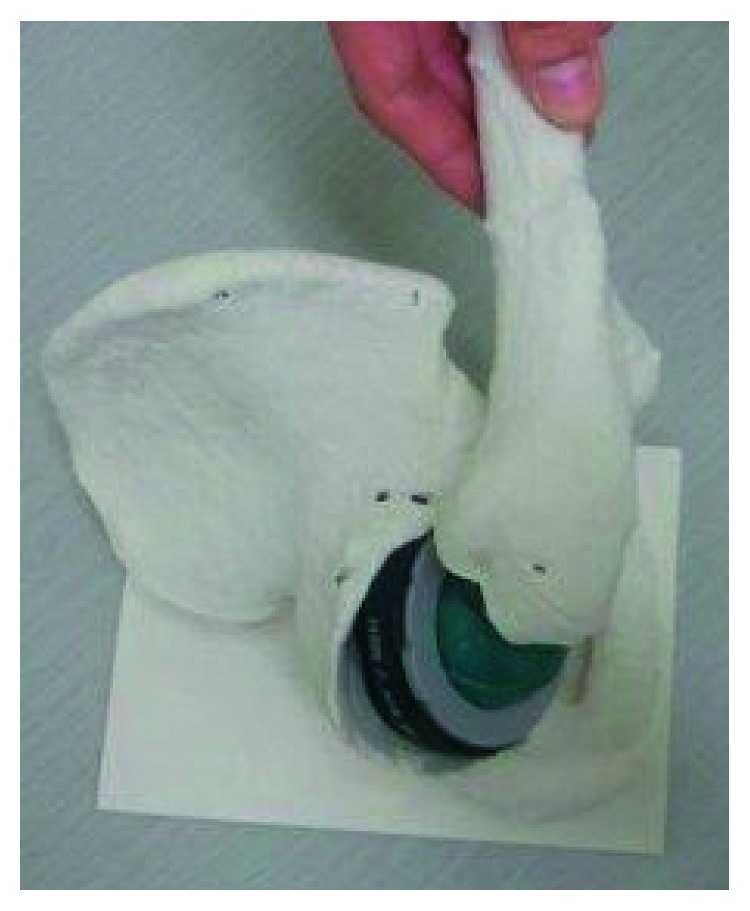
Simulation of surgery, using a 68-millimeter diameter acetabular shell, a tapered wedge stem, and a 44-millimeter femoral head. Bony impingement occurs between the anterior and anteroinferior acetabulum and the proximal femur.

Thus, bilateral THA was considered the treatment of choice. For planning, full-scale three-dimensional plaster models of the pelvis and femora were produced with an inkjet printer method using CT data (Next21 K.K., Tokyo, Japan). The acetabular cavity was aspherical, and the wall was thin even in the safe zone [13] (Figure 4). To fill the vault, the diameter of the acetabular component needs to be more than 66 millimeters. When a 68-millimeter diameter hemisphere was set at 0° of anteversion, its posterior part projected backward from the acetabulum, and its anterior part was deep in the bony wall (Figure 5). The goal was to gain good ROM without impingement. Thus, the plan was to fix a 68- or 70-millimeter diameter acetabular shell at 40° of abduction and 0° of anteversion in terms of the anatomical measurements [14], remove a significant amount of the bone from the anterior and anteroinferior parts of the acetabulum, use a 44-millimeter head, and fix the stem at 30° of anteversion (Figure 6). It was also considered vital to avoid reaming too deeply to avoid penetration of the acetabular wall.

The patient underwent right uncemented THA through a posterolateral approach. The acetabular component was a 68 mm, hemispherical shell (Trident, Stryker Orthopaedics, Mahwah, NJ, USA) with a highly cross-linked polyethylene liner (X3, Stryker Orthopaedics) and two transfixing screws. The femoral component was a tapered wedge stem with a neck shaft angle of 127° (Accolade TMZF, Stryker Orthopaedics) with a 44 mm (+4) modular head. These components were fixed at the same angle as at preoperative planning. Passive motion was possible from 30° to 90° in the sagittal plane.

Two months later, she underwent left THA through a posterolateral approach. The acetabular component was a 68 mm Trident shell with an X3 liner and 2 transfixing screws. The femoral component was an Accolade TMZF stem with a neck shaft angle of 132° with a 44 mm (+0) modular head. This time, the shell was placed at 35° of abduction and 10° of anteversion. Passive motion was possible from 30° to 100° in the sagittal plane. Although the surgery was uncomplicated, migration of the shell was found 2 weeks later. Thus, the unstable shell was removed, and a 70 mm Trident shell was placed with 6 transfixing screws at 45° of abduction and 10° of anteversion.

**Figure 7:**
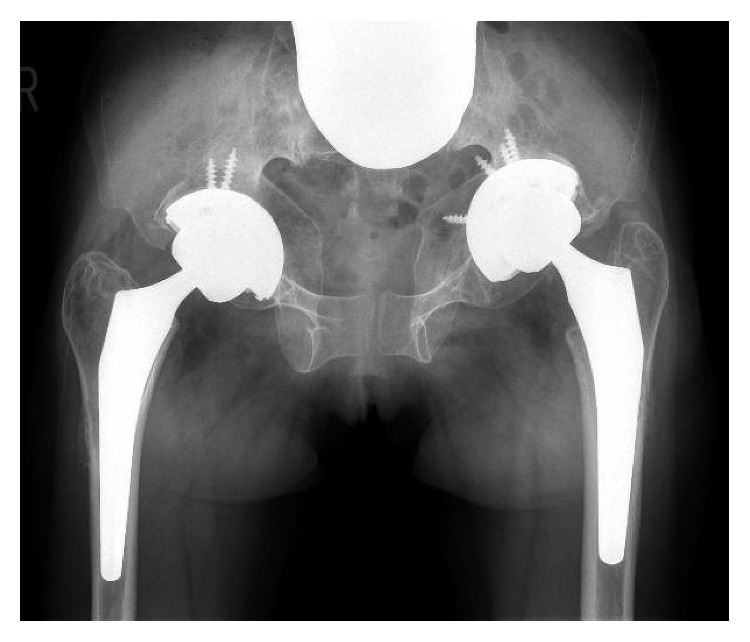
Postoperative radiograph of the hip joints, taken 1.5 years after the last surgery, demonstrating well-fixed components.

**Figure 8:**
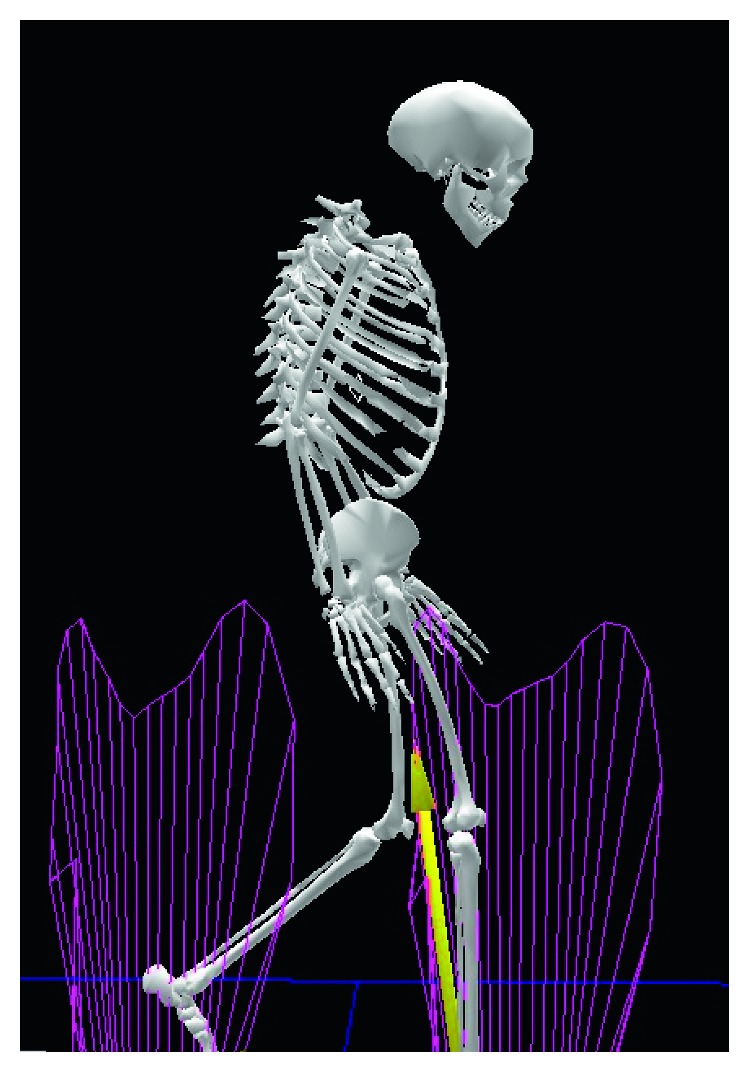
Postoperative three-dimensional motion analysis demonstrates that the patient's gait pattern has improved significantly.

At 1.5-year follow-up, the patient had no hip pain and could walk without walking aids for about 10 minutes. Even though the flexion contracture had remained in both hip joints after surgery, ROM improved with active exercise. Thus, passive ROM of the right hip showed 100° of flexion with no flexion contracture and 20° of abduction and that of the left hip showed 80° of flexion with no flexion contracture and 10° of abduction. The HHS was 74 on the right and 73 on the left. Radiographs demonstrated stable components (Figure 7). Three-dimensional motion analysis demonstrated that her center of gravity had shifted posteriorly because of the improvement of the flexion contractures of bilateral hip joints and that her gait pattern improved significantly (Figure 8).

## 3. Discussion

Recent advancements in computer technology have made it possible for orthopedic surgeons to use various methods for planning THA, including three-dimensional templating [15], solid bone models [16], and simulation surgery in virtual space [17, 18]. Moreover, three-dimensional motion analysis provides surgeons with objective assessment of the efficacy of THA [10, 11, 19]. Although these could be useful for patients with inherited skeletal disorders, only two cases have been previously reported [20]. Therefore, we use three-dimensional motion analysis to the patients with the complicated deformity in THA. The three-dimensional motion analysis may be difficult to predict the postoperative postural change; however, the information gained by the present case will give us the specific information about the posture in such patients suffering from skeletal disorders.

In the present case, arthritic changes of the knee joints and hyperlordosis of the lumbar spine were evident. To determine the indication for THA, a preoperative three-dimensional motion analysis was performed; it demonstrated that hip dysfunction had a major impact on gait disturbance. Therefore, positive results were anticipated with THA. Full-scale three-dimensional models provided useful information on the size and position of the prostheses, the depth of acetabular reaming, and the extent of the bone that should be removed to avoid bony impingement and gain good ROM. After the second THA, however, migration of the acetabular shell occurred in two weeks. The cause of this complication was probably insufficient anchorage of the shell. The follow-up three-dimensional motion analysis confirmed the positive results of THA.

Compared with simulation surgery in virtual space, three-dimensional models seem to be less sophisticated. However, the possible advantage of these models is that surgeons are able to place the actual implants on the models as a surgical simulation and grip a structural image of the skeleton [16, 21]. In particular, this model is useful for patients with skeletal dysplasia because unusual and severe skeletal deformities are common findings in these individuals.

In conclusion, based on the experience of the present case, three-dimensional motion analysis appears to be useful not only for postoperative evaluation but also for preoperative assessment, especially in patients with multiple joint disorders. In these patients, it is often difficult to identify the joint that should be treated first to alleviate gait disturbance. A three-dimensional motion analysis provides surgeons with scientific support for surgical intervention of the particular joint.
